# A gap between the philosophy and the practice of palliative healthcare: sociological perspectives on the practice of nurses in specialised palliative homecare

**DOI:** 10.1007/s11019-019-09918-2

**Published:** 2019-08-05

**Authors:** Stinne Glasdam, Frida Ekstrand, Maria Rosberg, Ann-Margrethe van der Schaaf

**Affiliations:** 1 Department of Health Sciences, Faculty of Medicine, Lund University, Baravägen 3, 222 41 Lund, Sweden; 2 Palliativ vård och ASIH Helsingborg, Skåne, Sweden

**Keywords:** Specialised palliative homecare, Philosophy of palliative care, Nurses, Bourdieu, Medical logic, Neoliberalism

## Abstract

Palliative care philosophy is based on a holistic approach to patients, but research shows that possibilities for living up to this philosophy seem limited by historical and administrative structures. From the nurse perspective, this article aims to explore nursing practice in specialised palliative homecare, and how it is influenced by organisational and cultural structures. Qualitative, semi-structured interviews with nine nurses were conducted, inspired by Bourdieu. The findings showed that nurses consolidate the doxa of medicine, including medical-professional values that configure a control-oriented, positivistic approach, supported by the organising policy for clinical practice. Hierarchically, nurses were positioned under doctors: medical rounds functioned as a structuring structure for their working day. They acted as medical assistants, and the prevailing medical logic seemed to make it difficult for nurses to meet their own humanistic ideals. Only short time slots allowed nurses to prioritise psychosocial needs of patients and relatives. Point-of-actions had high priority, added financial resources and ensured that budgets were allocated. Weekly visits made it possible for nurses to measure, control and govern patients’ drugs and symptoms which was a necessity for their function as medical assistants. The findings challenge nurses to take on an ethical point of view, partly to ensure that patients and their families receive good palliative care focusing on more than medical issues and logic, and partly to strengthen the nurses’ profession in the palliative field and help them implement palliative care philosophy in practice.

## Introduction

An estimated 65% of the dying population in Sweden, and other high-income countries, will require palliative care (Morin et al. 2017). Development of palliative services in the community is increasing. Studies show that many patients prefer to die at home. They show that palliative care in the community gives patients and their families higher quality of life and care. In addition, it is less expensive than the traditional combination of homecare and hospital care as a result of decreasing the number of hospital admissions, shortening the length of hospital stays, and decreasing the frequency of emergency room consultations (Bainbridge et al. 2017; Brereton et al. 2017; Gomes et al. 2013; Seow et al. 2019). This article focuses on nurses and their work in specialised palliative homecare in respect of the philosophy of palliative care. World Health Organisation (WHO) defines palliative care as: … *an approach that improves the quality of life of patients and their families facing the problem associated with life*-*threatening illness, through the prevention and relief of suffering by means of early identification and impeccable assessment and treatment of pain and other problems, physical, psychosocial and spiritual.* (WHO 2018). The philosophy of palliative care is based on a holistic approach to patients which aims to relieve and control pain and other symptoms and to improve the quality of care for patients and their families. Palliative care is regarded as patient-centred, comprehensive, and multidimensional, addressing physical, psychological, social, and spiritual dimensions of patients and their families (Wachholtz et al. 2016). Palliative care is ideally provided by multidisciplinary teams, which include doctors, nurses, psychologists, physiotherapists, clinical pharmacists, occupational therapists, dieticians, and social workers etc. (Brereton et al. 2017).

The role of nurses in palliative care is normatively described as relationship building, supportive, communicative and coordinating (Cameron and Johnston 2015). Sekse et al. (2017) carried out a systematic literature review to explore how nurses describe their role in providing palliative care for patients with life-threatening diseases. They found that nurses are in a role where they are available, which gives them a pivotal role in palliative care and paves the way for nurses to be coordinators of care for patients, relatives and other healthcare professions in the medical field. Consistent with palliative care philosophy, nurses describe how they work from a holistic perspective, handling a breadth of activities in their working day. This includes the requirement for them to be attentively present, dedicated and able to use flexible and non-traditional methods in their work. The role of nurse implies standing in demanding situations with a lack of time and resources (Sekse et al. 2017). Studies show that nurses find that they establish a one-to-one relationship with each patient and his/her family in palliative care, where the patient’s situation requires the nurse’s support and involvement (Parola et al. 2018; Sekse et al. 2017). Nurses find that teamwork stimulates reflection and professional progress promoting both individual well-being and team-building (Dalgaard and Delmar 2008; Klarare et al. 2013; Parola et al. 2018). Furthermore, working in palliative care leads to a confrontation with nurses’ own mortality (Parola et al. 2018).

In practice, a number of empirical studies show that the possibilities for living up to the philosophy of palliative care seem to be limited by historical and administrative structures (cf. Engström 2012; Dalgaard 2007; Glasdam et al. 2013; Karidar et al. 2016; Klarare et al. 2013). Specialised palliative homecare puts high demands on the nurse’s competence because symptom control and assessments move out of the hospital into the home environment, which means that nurses are challenged in balancing proximity and distance in the different roles of professional care-giver, guest and friend in the relationships with patients and their families (Engström 2012; Dalgaard 2007; Raunkiær 2007). Studies show that allocated time, resources, leadership and contexts influence nurses’ tasks, priorities and relations in the home of the patient and in the team (Dalgaard and Delmar 2008; Klarare et al. 2013).

Furthermore, studies show that doctors, social workers and nurses in palliative homecare spend most of their working day conducting patient administration. During their working day, they have minimal contact with patients and their relatives (Karidar et al. 2016; Karidar and Glasdam 2018). Different meetings and medical rounds, driven by a medical logic where diagnoses and treatments are in focus as the agenda for the inter-professional team, structure the professionals’ working days. Other studies show that hierarchies in the medical field are constructed on doctor-oriented and institution-oriented rather than patient-oriented practices, which means that psychological and social issues in patients and their relatives have low priorities in the teams’ work and collaboration (Glasdam 2007; Klitzman 2007). In specialised palliative care, professionals work in interprofessional teams (Klarare et al. 2013; Karidar et al. 2016; Karidar and Glasdam 2018), based on professional knowledge and skills, professional roles and identities, and power and status among the professionals (Baxter and Brumfitt 2008). The collaboration conforms to the hierarchical structure in the team, and the system of hierarchy makes it difficult for teams to achieve a high level of coordination and cohesiveness on issues unrelated to the doctors’ diagnosis and treatment (Baker et al. 2006).

Thus, there seems to be a gap between the philosophy of palliative care and the practice of nurses working in the field of palliative homecare. The aim of this article is to explore, from nurses’ perspective, the practice of nurses in specialised palliative homecare, and how this practice is influenced by organisational and cultural structures.

## Method

The study was based on nine qualitative semi-structured interviews of nurses in specialised palliative homecare in Southern Sweden, in autumn 2016. The analysis was inspired by Pierre Bourdieu’s concepts of field, habitus and capital (Bourdieu 1977, 1984, 1990, 1995, 1996a, 1998; Bourdieu et al. 1999). Bourdieu´s theoretical framework functioned as background to the interview process and to the analysis of the empirical material.

### Theoretical framework

Bourdieu explored how and why people act as they do in concrete social contexts, and therefore it is appropriate to reflect this theoretical perspective in our study. He constructed a theory of practice, including the theoretical concepts of capital, fields and habitus. Bourdieu takes into account the opportunities within the actor (person, institution or organisation) and, above all, the structure that characterises the situations in which the actors practice (Bourdieu 1977). Bourdieu (1977) showed that these objective relationships are not immediately available for the acting actor, but only through scientific work by which the objective relationships can be constructed and made valid. Bourdieu never gave a simple definition of his theoretical concepts but instead showed through his empirical studies how these concepts can be used as a tool for achieving a break with everyday thinking and a realistic notion of the social world (Borelius 1998; Broady 1991, 1998). His theory of practice explored the practice of the field, unconscious thought and action patterns of the actors, through the concepts of habitus and capital in conjunction with the logic of the field (Bourdieu 1996a). Bourdieu (1977) used the concept of doxa to denote that which is taken for granted in any particular society. Doxa is the experience by which the natural and social world appears as self-evident. It encompasses what falls within the limits of the thinkable and the sayable.

According to Bourdieu, a field is a theoretical construction that symbolises an arena of production, circulation, appropriation and exchange of goods, services, knowledge or status. Each field has its own set of positions held by actors who struggle to define, accumulate, exchange and monopolise different kinds of power resources. Such resources are unequally distributed (Bourdieu 1984, 1996a). In our study, we regarded specialised palliative homecare as a space in the field of medicine. Many studies showed how the logic of medicine, including palliative care, is based on an individualistic, bio-medical rationality, in which the diagnosis is foundational for making valid interpretations of the patient’s somatic condition and accurate decisions about his/her treatment (Glasdam 2007; Mol 2008; Engström 2012; Højskov and Glasdam 2014; Glasdam and Oute 2018). It has also been shown that the logic of medicine formed an organising policy for clinical practice. Moreover, at the same time, the logic of medicine constituted professionals’ identities, autonomies and positions within a particular field (Glasdam and Oute 2018).

This must be seen in connection to habitus, understood as the construction of the practical sense of the actor, built up through family history and the lived life. Persons have an orientation—a practical sense—that produces outlines of discourses and practices. Habitus consists of the ways in which the socio-symbolic structures of society become deposited within persons in the form of lasting dispositions, trained capacities and patterned propensities to think, feel, and act in certain ways (Bourdieu 1990, 1995, 1996a, 1998, Bourdieu et al. 1999). According to Macintosh (2009), habitus is the logic or code for the social behaviour of a field. The position of actors in the field is determined by the amount and importance of the capital. There are four types of capital: (1) economic capital, such as money, property and other valuable assets; (2) social capital, such as relationship resources, friends and family; (3) cultural capital, such as education and job and; (4) symbolic capital, such as the person´s status, prestige and honour (Bourdieu 1977). Bourdieu always thinks in relational perspectives, characterised by an interest in what is happening between people when they interact in social space. Relational concept means the analysis of the relation between social positions and dispositions (Bourdieu 1984, 1996a). In our study, we explored how the nurses’ habitus and capital, the logic of medicine and the structural, political and administrative frameworks constituted, framed, directed and enabled nurses to think and act in specialised palliative homecare.

### Recruitment

The study took place in three public, specialised palliative homecare services in Southern Sweden. Inclusion criteria were that informants had at least 2 years of working experience in specialised palliative homecare, and were employed as nurses. There were no exclusion criteria. The senior managers of specialised palliative homecare services approved the project. At workplace meetings, unit managers informed the nurses orally about the study and provided a contact to put interested nurses in contact with researchers. Ten nurses wanted to participate in the study, but one was excluded because of short work experience. All nine participants were informed about the aim of the study both orally and in writing by the interviewer, and they gave informed consent. All participants were women between 41 and 64 years old. Professional experience as a nurse varied from nine to 37 years. Five nurses were educated to a specialised level, and four had basic nurse education.

### Interview process

A semi-structured interview guide with three themes was constructed to support the interviews, inspired by Bourdieu’s theoretical concepts. The first theme concerned the sociological background of the nurse and aimed to create a picture of the person in order to analyse her capital, habitus and social position. The second theme addressed the content in her working day based on descriptions from a concrete day. The third theme concerned the nurse’s relations to other persons in her working day based on descriptions from a concrete day. The second and third themes created an in-depth picture of the person’s position, content of the working day, the relations and the workplace, and the prevalent values, norms and rules. Descriptions of what the nurse did and with whom may also show the capital and habitus that nurses have at the workplace.

Three interviewers did three interviews each. All interviews were in Swedish, and lasted 40 to 105 min with an average duration of 82 min. The interview persons decided where interviews were held, in order to create a safe and comfortable framework for them (Glasdam 2005). A pilot interview was made and transcribed in order to test the interview guide, train interviewers in interviewing and align the interviewers’ techniques and attention points in relation to each other (Bourdieu et al. 1999). The pilot interview initiated discussion of interviewing, interviewing techniques and attention points, such as use of silence and patiently waiting for the informant’s stories in the interview situation. No changes were needed in the interview guide. The pilot interview was not included in the empirical material. The interviews were recorded on a Dictaphone.

### Ethical considerations

The study followed the principles of the Helsinki Declaration (World Medical Association 2013). It has been approved by the Advisory Committee for Research Ethics in Health Education (VEN) at Medical Faculty, Lund University (J.No. VEN 82-16) and is in accordance with Swedish legislation. The researchers informed all interested nurses, orally and in writing, that all participation was voluntary and that they could withdraw at any point without explanation. Informed consents were obtained.

### Analytic strategy

A latent content analysis, inspired by Bourdieu’s theoretical framework, was made and inspired by other healthcare studies using Bourdieu as a theoretical lens (Engström 2012; Glasdam and Øye 2014; Glasdam and Oute 2018; Karidar and Glasdam 2018; Angus et al. 2018). First, all interviews were transcribed verbatim by each interviewer. Second, the empirical material was read through in its entirety several times by all four researchers to get an idea of its contents and an overall picture of all the interviews. Third, the transcribed interviews were analysed, initially one by one and then across the entire empirical material. A theoretically inspired analysis was conducted, focused on how the nurses articulated work in specialised palliative homecare. Bourdieu did not specifically advise how to perform analysis. It must be regarded as a creative process (Glasdam et al. 2015). Bourdieu’s concept of field, habitus and capital functioned as a perspective from which the empirical material was read, understood and interpreted (Glasdam 2005, 2007). A matrix was constructed for each interview, horizontally containing information about: facts, viewpoints on facts (positioning), and how to act. The vertical column contained information about: who the person was from a sociological perspective, what s/he did on a working day, whom s/he met in a working day and what they did together. Quotes from the interview text were placed in an associated column. Each interview had 8–16 pages of the matrix, which helped to establish an overview of the empirical material. Next, all interviews were analysed across the interviews, and themes and subthemes were constructed, see Table 1. *Quotes* were selected from the empirical material to serve as illustration for the analysis.

**Table 1:** Themes and subthemes

Subtheme	Theme
Nurses as medical assistants The medical round as a structuring structure for nurses’ working day	The logic of medicine ruled nurse practice
Organisational, financial earning opportunities governed nurse actions Planned weekly visits as structuring structure for controlling and conducting treatment	The organisational structure ruled nurse practice

## Findings

### The logic of medicine ruled nurse practice

#### Nurses as medical assistants

Through their professional experience, nurses established a clinical gaze, a feeling or experience that was difficult to define. They called it an intuition or ‘gut feeling’, which was important to listen to. The nurse’s professional habitus developed with experience and they learned how to act in different situations by experiencing intuitive or gut feelings or through theoretical knowledge and education. Bourdieu calls it the practical sense, that is, how the nurse acted instinctively.

After many years working experiences, you realise that you have a lot of intuition, which is a good feeling. I do not know if that’s what you pass on from novice to expert but you should be aware of and acknowledge these intuitions. (Nurse 7)

A medical gaze primarily ruled these intuitive actions. The medical logic seemed to govern the nurses’ practical sense and her resulting actions.

There was no pain or anything like that, but the patient was anxious, and so I said “I’ll be here again in half an hour” and they accepted this. The patient - who was relatively new in specialised palliative care - got his diagnosis at a rather late stage, he felt unwell but could not find out the cause, and he was worried. He did not want any drugs. I spoke the doctor. I said there’s something wrong, but I don’t know what it is - should we take blood samples so we have some starting value? Yes, do it, said the doctor. (Nurse 8)

Nurses described their tasks at patients’ homes. These were often medical, and nurses felt comfortable with them. The nurses faced problems when there were: changes in a patient’s cancer or condition so that the current medical plan was inappropriate’; ambiguities in the medical plan for the patient; or when the medical plan did not work as intended. In these situations, the nurses would contact the doctor for a new medical plan. The doctor provided direct advice to nurses about alternative medical strategies, so that nurses could decide on a strategy in dialogue with patients and/or relatives.

Then [because of deviations from the expected situation], I had to contact and talk to the doctor. We put a plan together, the doctor said what I could say, what alternatives were there, what I could say to the relatives. (Nurse 4)

Hierarchically nurses were positioned under doctors in specialised palliative homecare. Nurses’ habitus was characterised by the medical logic and they saw themselves as the extended arm of the doctors. Nurses in the subfield had also a relatively high educational (cultural) capital due to their training in palliative care as compared with municipality healthcare professionals. In Sweden, the municipality healthcare professionals provide general palliative care to patients such as personal hygiene, uncomplicated wound care, dispensing of the medicine, et cetera, where professionals in specialised palliative care are responsible for patients with complex symptoms and special needs (Regionala cancercentrum i samverkan 2016).

The patient is not happy that she will be discharged [from specialised palliative care]. It’s an older lady who will not die from this tiny tumor she has. She has no [tumor-related] symptoms to be relieved. It’s an old woman who is tired because of old age and what she needs is a homecare nurse who can dispense her medicine and general care. It’s the wrong level of care to have her included in specialised palliative care. (Nurse 3)

This position gave nurses symbolic capital in other work situations, such as a higher position when cooperating with other professionals around the patient. Nurses in specialised palliative homecare had a mandate to train municipality staff and delegate tasks regarding patient care and drug administration at home. From the nurse perspective, collaboration with other healthcare professionals was a challenge, and they described their function as a consultant regarding end-of-life care.

There’s a lot of interaction and it’s important. It doesn’t always work. Because, I think, palliative care isn’t palliative care, if it doesn’t work all the way to the bedside situation, and we don’t have the power over the bedside situations. We are dependent on the municipality staff and we need to have a good dialogue with them. […] I train them or at least act as a consultant to them, so I would like to have much more meetings with the municipality staff. (Nurse 7)

Nurses in specialised palliative homecare served as intermediaries between nurses in the municipality and doctors, when they communicated information, medical plans and prescriptions.

It’s still us [specialised palliative homecare] who implement the decisions [about medical plans for patients]. I had talked to the doctor about it, he told me that if he’s [the patient] bad again, cancel [treatment]. (Nurse 1)

We have the medical responsibility and they [municipal homecare] have the nursing care responsibility. (Nurse 8)

Often, other healthcare professionals were involved with patients. Nurses in specialised palliative homecare were positioned relatively high in relation to other caregivers and had tools to gain an overview and control of patients’ illness and tasks related to them.

We have SIP, coordinated individual planning, which we have to do when there’s more than one caregiver and that’s great because then it’s in writing who’s doing what. (Nurse 7)

Nurses in specialised palliative homecare were positioned hierarchically under the doctors and over the municipality’s nurses in terms of medical responsibility. Nurses handling of and responsibility for medical treatment also seemed to have more symbolic capital than care in specialised palliative homecare, which also showed the priority of the nurses: medicine and medical tasks first. From nurse perspective, care was primarily a task for professionals in municipal homecare.

When nurses described their working day, they immediately had a few patient visits and temporarily a limited direct contact with patients and their relatives at home.

Currently, there’s this course in the IPOS [Integrated Palliative Care Outcome Scale] […..] There’re several smaller things in the working day that steal time […] After all, it the patient’s time that is cut short from it. (Nurse 2)

[In the mornings,] we look through our schedule and […].find out how to distribute it [the work] between us [the nurses], so we can make it a good day. I had two [patient] visits, and the doctor was with me that day. (Nurse 3)

Nurses were often travelling in the car or were at the office for indirect patient work such as medical rounds, report, administrative duties and collegial mingle. Nurses articulated a desire to be closer to patients in order to optimise control of situations and make their own assessments. Nurses had to rely on assessments of patients’ symptoms made by relatives or municipality healthcare professionals.

I’m so reliant on their assessment [relatives and other healthcare professionals], that’s fine, but it’s much better if I can see it myself…. And if I could get just 20 min there [at the patient’s home]. (Nurse 7)

This contrasted with how nurses described their job in general terms in specialised palliative homecare. In their general descriptions of their job, nurses highlighted concepts such as psychosocial focus, communication and openness about disease and death, and relational attention. This could be regarded as originating in the educational (cultural) capital that nurses had through their education where they were taught that a humanistic perspective regarding patients was important in their relational profession. Those highlights seemed important for the understanding of nurses’ role and function in specialised palliative homecare.

I like the psychosocial, the conversations and yes, that you see the whole person and family as we do here [in specialised palliative homecare], the whole. Not like at a surgical ward, just meeting the patient. (Nurse 1)

So it’s outstanding to be and have such a job where you can relieve and help, and it doesn’t have to be medical stuff, but it can also be conversational if you have time to stop and talk and you feel they are satisfied, and that they feel safer now than when I arrived. I think it’s amazing. (Nurse 7)

In nurses’ descriptions of what they did in their working day, there were limited situations where care for patients and support to relatives were a part of their practice. Some nurses used unplanned timeslots, which emerged for spontaneous visits to patients and support of relatives. As a result, they were able to retain the view that their work in specialised palliative homecare had a humanistic basis in patients and their relatives.

If we had been busy, I wouldn’t have done that. Now I think it should be a spontaneous, unannounced visit. It doesn’t take me many minutes. Relatives do not always see the patient’s pain, anxiety, or anything else. There can be so much we can help with, as they don’t notice. (Nurse 8)

You don’t ask [whether relatives wish us to visit], and maybe they don’t say anything. I still think we’re there for them, and if they want [to have a visit], it’s clear that we will come. But when I talked to colleagues, they say ‘I never ask that question’. (Nurse 1)

At an administrative, structural level, it was legal not to include relatives’ needs for support. It depended on the individual nurse and at same time, there was a potential conflict between nurses. To a certain extent, nurses who prioritised relatives’ needs for support challenged the doxa and the logic of medicine in the subfield. It seemed to be difficult for nurses to meet their own humanistic ideals in specialised palliative homecare, where the medical logic was prevalent and only unexpectedly emergent time slots allowed nurses to prioritise psychosocial needs for patients and relatives.

#### The medical round as a structuring structure for the nurses’ working day

From the nurse perspective, the medical round appeared as a structured structure, organising and governing the nurses’ practice in specialised palliative homecare. A doctor and a varying number of nurses carried out the medical round. The nurses’ task was to go through the blood tests, drug list and each patient’s medical planning with the doctor. The medical round was also an opportunity for dialogue and updating about the patient’s status. Nurses’ actions during the round consisted of communicating information and asking questions about the patients. Furthermore, the medical round added tasks for nurses, such as planning of blood samples.

Then we took the patients one by one and we went through everyone, looking at what’s the plan in the near future, is there something we have to fix, the doctor looks over the blood samples as “It’s been a long time since we have taken blood samples; we can take them at the visit next week”. And I had the calendar and noted everything agreed, and [the doctor] wrote the referral - so it became real teamwork. (Nurse 9)

We have medical rounds. We go through almost everything: Lab lists, medicine lists, what should we think about, what’s the plan and so forth. [The other days, the doctor and the nurse] briefly go over the patients that have something that need to be discussed […], they just scan the patients quickly, for example if they have vomited, if they have been treated and so on. Then the doctor informs me if there is something I should observe or know. (Nurse 5)

Nurses’ habitus was a product of the socialisation that had taken place both during education and through work in the medical field, where nurses were educated to ask the right questions, and to communicate relevant information to the doctor knowing her function and position in the medical round.

The medical round created consensus between nurse and doctor regarding the patient’s future medical planning. This planning governed what was important to prioritise, pay attention to and how nurses could and should act in relation to patients from a medical perspective. In specialised palliative homecare, doctors were hierarchically positioned relatively higher than nurses and close collaboration with doctors was a valuable symbolic capital for nurses, from nurse perspective.

Well, now we’ve gone through the patients, you’ve an overview of every single patient and you feel more like a team when you agree with the doctor, you hear her reasoning because she knows things that we don’t know, and we see patients more than she does. (Nurse 9)

In the medical round, teamwork meant creating a situation where doctors got the information from nurses they needed to treat the patients, and the nurses got the knowledge to be able to assist doctors in their work with patients. From the nurse perspective, the medical round was important, for both doctors and nurses, and ruled most of the nurses’ actions in a working day.

### The organisational structure ruled nurse practice

#### Organisational, financial earning opportunities governed nurse actions

Specialised palliative homecare is organised as part of public, advanced homecare in Sweden. This means that nurses also work with advanced healthcare at home on behalf of other clinics, and have medical point-of-actions, understood as short-term medical treatment, for both palliative and non-palliative patients of all age, e.g., blood transfusion and intravenous antibiotic treatment. In the study, the homecare service was solely responsible for these medical point-of-actions, while the referring unit had primary responsibility for patient care and treatment.

I didn’t really put myself into it so much […]. You don’t have to read the entire history of disease, but this is a prescription from the patient’s surgeon, fine, so then we’ll do it. (Nurse 7)

These point-of-actions added to the homecare institutions financial resources and ensured that budgets were held. Therefore, they had the highest priority of tasks in specialised palliative homecare. At the same time, these actions required that nurses acted as doctors’ assistants and conducted their prescribed treatments.

They [point-of-actions] will not be put in any queue or in any ring binder because they will be executed within a few days. (Nurse 5)

The point-of-actions patients are registered in WebPasis, because it’s the referring clinic that pays those visits, so it’s very important. (Nurse 9)

According to nurses, the referred point-of-actions were only medical tasks; psychosocial attention to the patient was not included in the price.

They [point-of-actions patients] don’t have anything to do with specialised palliative homecare, but, of course, you sit down and talk to him [during treatment]. (Nurse 4)

You hardly ask the patients how they feel when you work in the point-of-action team. (Nurse 2)

It was not considered important which nurse was traveling to point-of-action patients, only that someone went. It meant that economic capital had a relatively high priority in specialised palliative homecare, and that point-of-actions to—in principle, all type of patients—had a higher priority and value than patients receiving palliative care in this subfield of medicine.

#### Planned weekly visits as structuring structure for controlling and conducting treatment

Every week, all patients receiving palliative care were meant to receive a nurse visit, at which the nurse would deliver drugs and other material to the patient, and measure the patient’s symptoms with different tools. Through nurse education and professional socialisation, they were disposed to have properties such as control and overview incorporated in their habitus, and they found it natural to prepare these visits carefully in order to establish control over patient meetings.

I read in Melior [a journal system], the latest status, the history and something else written in the journal. Then I pack and take drugs that I should have with me. That’s what I think I’ll need, or what I know I need. (Nurse 6)

The weekly visits made it possible for nurses to control and govern the patients’ drugs as a necessity for their function as medical assistant. At the same time, they disciplined patients to fit into logic of the organisation.

Previously, they [patients] have been able to pick up 200 tablets from the pharmacy, and then we come with seven and it’ll be enough until next week. [….] ‘I have one tablet left, can you come now?’ and ‘No, we cannot?’ It’s a lot about being forward-looking. We must teach them [patients] to call on time. (Nurse 7)

The weekly routine assessments of patients’ pain or other symptoms as well as oral status were part of an organisational quality assurance of healthcare, regardless of the patient’s needs or situation. Nurses documented the assessments in the journal.

In Melior [journal system], we have a planning system, showing exactly what to do during the weekly visit. We do something called ESAS [**E**dmonton **S**ymptom **A**ssessment **S**ystem] […] We do oral status every week, pain assessment in general, every week. (Nurse 9)

These weekly visits, both structurally planned and structurally orchestrated at a management organisational level, were considered to provide quality assurance of healthcare in specialised palliative homecare. Nurses explained that the management regularly displayed statistics of the nurses’ measurements and used these as a signal for quality of care and attention towards patients.

When measuring, you know that the patients have been seen. (Nurse 8)

Tacitly, nurses disciplined patients to be passively subordinate to the frame and logic of the field of medicine. This study focussed on the subfield of specialised palliative homecare, which showed a power structure where patients were positioned hierarchically under professionals. Patients’ need for drugs and nurses’ administration of the drugs increased patients’ dependence and consolidated patients’ subordinate position in specialised palliative homecare. Thus, nurses socialised patients into the subfield on the subfield’s premises and it turned out that patients, according to nurses, learned what was expected during the weekly visit.

It’s actually a good parameter, and now it’s routine, so you don’t think about it, and the patients, they almost stretch out the tongue when we come. Do you have any problems with your mouth? Ah’ [stretches out her tongue]. They understand why if you explain some things. (Nurse 9)

Overall, from the nurse perspective, the weekly visits largely structured nurses’ actions before, during and after the visits. Every nurse was primarily responsible for a number of patients, in which role she was called ‘contact nurse’, and it was very important for her to obtain the right amount of knowledge about the patient and relatives in order to screen problems and plan actions.

And then I know, they know who I am, and what I’ve been able to do, and I feel instantly that they trust me. (Nurse 1)

Nurses prioritised contact nurse visits higher than visits to other patients, and being a ‘contact nurse’ became symbolic capital for nurses.

She had the desire to go to two patients whom she hadn’t met for a long time, and then I took […] the other visits. (Nurse 9)

The organisational structure in the subfield could be both supportive and obstructive for nurses in the nurse’s contact system. It was supportive in that the nurse was responsible for a certain number of patients and was encouraged to establish good relationships with them.

Since I’m going to work for a couple of days in a row, I’ll drive there today. (Nurse 6)

It was obstructive in that organisational conditions such as lack of staff meant that continuity could not always be prioritised.

## Discussion

The study shows how the logic of the medical and organisational structures creates difficulties and challenges the opportunities for nurses to work within a frame of the philosophy of palliative care. Nurses express a predominantly medical orientation when they talk about their work and working days in this study. Nurses are socialised into the role of assistants to doctors, and the medical logic unconsciously rules their practical sense in specialised palliative homecare. It is important to be aware of how, historically, nursing is embedded in the medical field, which has significance for the context in which nursing is carried out in palliative homecare and the conditions under which nurses perform their jobs (Glasdam 2007; Carlhed 2007; Glasdam and Oute 2018). According to Bourdieu (1996b), an acquired habitus maintains an order that classifies people and knowledge. Through a magical power, certain people, institutions and titles are recognised and acknowledged as honourable, superior or reverent. This recognition and acknowledgement is not an individual matter, but is based on group beliefs and is linked to the power structures of society. This study shows that medical knowledge and skills are relatively high cultural and symbolic capital for nurses. Although nursing and caring are regarded as important, medical tasks appear to be more important and have a higher capital value than caring. Different meetings and medical rounds, driven by a medical logic where diagnoses and treatments are in focus, structure nurses’ working days. They spend most of their working day conducting administration on behalf of, and with, patients and colleagues and driving between meetings. Actually, they have little time with patients and their relatives, as also shown by Karidar et al. (2016). In a study of nurses in Danish homecare Ellegaard and Dybbroe (2015) show the same pattern that homecare is ruled by the medical doxa. Furthermore, they show that patients and relatives also expect that nurses should be medically competent and have good medical knowledge, a vision shared by society at large (Ellegaard and Dybbroe 2015). The philosophy of palliative care has difficult conditions within this historical medical doxic thinking, when it appears that addressing psychological, social, and spiritual dimensions of patients and their families (Wachholtz et al. 2016; WHO 2018) is prioritised lower than the physical dimension of the patients.

The study also shows how nurses in specialised palliative homecare support and consolidate the doxa of medicine, including the medical-professional values that configure a control-oriented, positivistic approach, which also form an organising policy for clinical practice. Glasdam and Oute (2018) find the same pattern in the oncological and psychiatric field. Furthermore, the study shows how the organisational frame in palliative homecare is closely connected to a neoliberal ideology, where the healthcare service works within an organisational system of cost saving procedures, documentation and efficiency (Eliason 2015). The results point to a neoliberal organisational frame in relation to the weekly measurements and documentation of the patient’s condition as well as statistical calculations thereof to assure and control quality of care within specialised palliative homecare. Moreover, the results point to this frame in relation to how specialised palliative homecare prioritised earning possibilities where point-in-action tasks were weighted at the expense of the palliative patients in the institution. Today, there is focus on cost-effectiveness and utility of public services, where the efforts also must be evidence-based (Glasdam et al. 2015). Rationalisation, quality management and control are key concepts in evidence-based practice (Olesen 2007). Evidence is an element of a growing management thinking that characterises the management of the healthcare system (Lihme 2015; Olesen 2007), since evidence-based practice is often about what works and does not work cost-effectively (Oeye et al. 2015; Wallin 2009). Focus is placed on goal management through financial goals and results follow-up (Damm 2014). The neoliberal organisational frame can explain the priority of point-of-actions in nurses’ working day; it is easy, it is fast, it requires a minimum of resources, it focusses on one task and one dimension of the patient, and it adds economic capital to the system. On the other hand, the neoliberal frame is incompatible with the philosophy of palliative care and the idea of a holistic perspective. It takes time to build trust in relationships, and it requires both time and trust to support people with psychosocial and spiritual issues and problems. In a neo-liberal framed organisation, time is understood as money (Martinsen 2009), which is why a minimal time spent is desirable, so that money is not wasted in the organisation.

The neoliberal organisational frame can also explain the nurses’ weekly measurements and documentation of the patient’s condition regardless the needs of the patients. Nursing care, techniques and treatments are transformed into evidence-based protocols, and procedures are measured and controlled through several communication and monitoring strategies (Krol and Lavoie 2014; Graven and Timm 2019). For example, The National Board of Health and Welfare (2006) in Sweden created a national quality register in 2005, the Swedish Palliative Register. It is a national quality register where the healthcare provider records the care of a person in the terminal stage of life. The purpose is to improve care at the end of life regardless of diagnosis and healthcare profession. Healthcare professionals answer a questionnaire with circa 30 questions about how the care has been during the last week of life. The idea is that healthcare professionals can use the results to see what quality the care holds and what needs to be improved, and when improvements are made. Results from the Swedish Palliative Register can be used for follow-up. The questions are about symptom relief, measurement of pain, documented oral health assessment, use of pharmaceutical injections for different symptoms, etc. (National Care Program for Palliative Care 2016). All the mentioned parameters control nurses’ practice according to this register. Therefore, it can be discussed whether nurses’ weekly routine visits to patients are an organisational structure based on the design of the registry or are actually based on patient’s physical, psychosocial or spiritual needs. The weekly use of measurement tools seems to affect professional practice and people’s everyday living on an individual level (Martinsen 2009; Blomgren and Waks 2010; Glasdam and Munksgaard 2016). However, measuring tools do not always imply new knowledge. They rather deliver contemporary valued means to create ‘hard facts’, e.g., by converting clinical practice into numbers (Sudmann and Glasdam 2019). Nilsson and Person (2014) show how these numbers replace what professionals have already registered through their bodily senses. A selective view on the results of an assessment might lead to a (non-holistic) reduction of human beings to a generic phenomenon void of culture, religion, gender, age, social class etc. (Glasdam et al. 2015; Martinsen 2009).

As shown in the study, it is difficult for nurses to meet their own humanistic ideals in specialised palliative homecare, when the medical logic is prevalent and only unplanned time slots allow nurses to prioritise psychosocial needs for patients and relatives. According to Bourdieu et al. (1999), the neoliberal ideology produces suffering for both the professionals working under such conditions, and the people exposed to action, care and treatment within such organisational neoliberal frameworks. Some nurses provide quiet resistance and challenge the medical logic and the organisational structure by spontaneous, unplanned home visits to meet their own and the philosophy of palliative care’s humanistic ideals for nursing and care. Through civil disobedience, nurses feel that they succeed in their job. Kennett (2017) argues that civil disobedience occurs if professionals think that aspects of ordinary healthcare practice involve them in serious moral wrongs that are not outweighed by considerations of the autonomy of patients or the running of the system: then conscience would often require that they challenge the borders of the possible. Henriksen and Vetlesen (2013) show that care and nursing cannot be priced, and through demands for efficiency, nurses are required to act against their ideals and norms. Nurses’ caring and relationship-creating habitus is most visible in nurses’ thoughts *about* their work. In concrete situations, it is nurses’ assistant and compliant habitus, according to medical logic and organisational framework, which is most prominent. Clearly there is a difference between what nurses imagine they should do and what they actually do, as has been also shown in other studies (Ellegaard and Dybbroe 2015; Karidar et al. 2016; Karlsson 2008; Oute et al. 2018). It is possible that ‘the holistic approach’ to palliative care is implicit in all actions by nurses in relation to palliative patients and their families, but it seems problematic that nurses cannot verbalise this part of nursing and thus describe it. It is difficult to describe the complexity of actions in palliative care and to put words on what holistic palliative care involves for nurses and their work. It may be difficult to capture such activities in the medical language. This can lead to significant parts of palliative care remaining invisible and thus not being prioritised—or documented. This challenges nurses to take on an ethical point of view, partly to ensure that patients and their families receive good palliative care focusing on more than medical issues and logic, and partly to strengthen the nurses’ profession in the palliative field and help them implement palliative care philosophy in practice. Graven and Timm (2019) echo this necessity of both articulating and prioritising the ethos of palliative philosophy in palliative care practice in their study of hospice philosophy in practice.

Finally, the limits of this study should be discussed. A weakness of the method is that it is based on how nurses articulate their practices. The study does not address how clinical practice is handled within practical reality. Likewise, the study does not address the perspective of other healthcare professionals, patients and relatives, which means that it is not possible to describe all the relational understandings of the complex reality and meetings in this study.

## Conclusion

The study show that it is difficult for nurses to meet their own humanistic ideals and the philosophy of palliative care in specialised palliative homecare, where the medical logic and the neoliberal organisational framework are prevalent and only unplanned time slots allow nurses to prioritise psychosocial needs for patients and relatives. Nurses supported and consolidated the doxa of medicine, including the medical-professional values that configure a control-oriented, positivistic approach, also supported and formed by the organising policy for clinical practice. Hierarchically, nurses were positioned under doctors, with medical rounds functioning as a structuring structure for their working day. Primarily they acted as medical assistants. Point-of-actions had high priority, added financial resources and ensured that budgets were held. Weekly visits made it possible for nurses to measure, control and govern the patients’ drugs and symptoms as a necessity for their function as medical assistants. Some nurses provide quiet resistance and challenge the medical logic and the organisational structure by spontaneous, unplanned home visits to meet their own and the philosophy of palliative care’s humanistic ideals for nursing and care. Through civil disobedience, nurses feel that they succeed in their job. By highlighting nurses’ practice, it is possible to reflect on how the palliative care and nurses’ role and function in specialised palliative home care can and should be in the future. This concerns the individual nurse, leaders at management and administrative level, healthcare professionals in practice in general, and actors at the political level in the society. Further, this study calls for an observation study investigating interactions between professionals, patients and relatives, exploring how clinical practice is handled within the practical reality from a relational perspective.
